# Supplementary material for: Heat shock factor 1 directly regulates transsulfuration pathway to promote prostate cancer proliferation and survival
Source: Commun Biol. 2024 Jan 3;7:9. doi: 10.1038/s42003-023-05727-9 (PMC10764307; doi:10.1038/s42003-023-05727-9)
Supplement: Supplementary file 2 — Supplementary Information [file 42003_2023_5727_MOESM2_ESM.pdf]

Hauck et. al. Supplementary Tables

Supplementary Table 1: HSF1 binding sites in Erythroleukemia Cells after Heat Shock

Gene(s)	Protein	Location in Gene	-10* log10(pvalue)	Fold Enrichment	False Discovery Rate%
HSPA1L, HSPA1A	HSP70	Promoter, Both	3044.89	25.55	0
HSP90AB1	HSP90	Intron	2177.38	23.75	0
CBS	CBS	Intron, Intron, Intron	573.09	10.99	0.45

Promoter: HSF1 binding site in the promotor of gene, Both: HSF1 binding site in the intron/ exon junction of gene, and Intron: HSF1 binding site in the intron of gene.

P value transformation reference: -10*log10(0.05)= 13.01 and -10*log10(0.0001)= 40.00

Supplementary Table 2: Vectors

Vector	Type	Sequence
pLKO-Tet-On-shRNA-Control	No template control shRNA	ATCTCGCTTGGGCGAGAGTAAG
pLKO-Tet-On-shHSF1 7480	HSF1 knockdown a	GCAGGTTGTTCATAGTCAGAAC
pLKO-Tet-On-shHSF1 7483	HSF1 knockdown b	GCCCAAGTACTTCAAGCACAAC
pLKO-Tet-On-shCBS 45359	CBS knockdown	GCGGAACTACATGACCAAGTTC
pCW-Cas9 NTC	No template control for knockout	GTATTACTGATATTGGTGGG
pCW-Cas9 KN200314G1	HSF1 knockout 1	GGTGTCCGGGTCGCTCACGA
pCW-Cas9 KN200314G2	HSF1 knockout 2	CGAGGGTCCACAGCTTGGTC
pCW-Cas9 KN401755G1	CBS knockout 1	TCTGCCTGGGGGGTCTCAGA
pCW-Cas9 KN401755G2	CBS knockout 2	GAAGGGGTCCCCAGAGGATA
pLV-U6-(NTC gRNA) 1800	No template control for steric inhibition	GTATTACTGATATTGGTGGG
pLV-U6-9(CBS gRNA 1) 1796	Steric inhibition of CBS 1st intronic HSF1 binding site 1	CTTTCTGGAAGATTCGGCGG
pLV-U6-(CBS gRNA 2) 1797	Steric inhibition of CBS 1st intronic HSF1 binding site 2	CCGAATCTTCCAGAAAGCCG
pLV-U6-(CBS gRNA 4) 1799	Steric inhibition of CBS 3'UTR HSF1 binding site	TATATGAAATGTCCAGAACA
pSTV2.0 GFP	CBS Overexpression	CBS ORF NM_000071.3<br>F Primer: GTCGCCGTCTCCGCC<br>R Primer: TTGTGTGATTCCATTTATAT

Vectors to genetically modify gene expression

Supplementary Table 3: qPCR and ChIP-qPCR Primers

Gene/ region	F Primer Name	F Primer Sequence 5'	R Primer Name	R Primer Sequence 5'
CBS	CBS_F	TCGTGATGCCAGAGAAGATG	CBS_R	TTGGGGATTTCGTTCTTCAG
HSF1	HSF1II_F	GCTGATGAAGGGGAAGCAGG	HSF1II_R	GACGACTTTCTGTTGCTGGG
ACTB	ACTB2_F	GCCGCCAGCTCACCAT	ACTB2_R	AGGAATCCTTCTGACCCATGC
CTH	CTH_F	CCAGCACTCGGGTTTTGAAT	CTH_F	AACCTGAAGCAAAGGCCAAAC
HSP70 Promotor	HSP70_ Prom_F	GGGGTGAGGTTAAGGGTCTC	HSP70_ Prom_R	GCTCCCTAATGAATCACCTCTC
HSP90 1st intron	HSP90_ 1i_F	TTCTGTGAGGGAGGGTTGAC	HSP90_ 1i_R	CCAGAACAATCTCAGCTCTCG
CBS 1st intron	CBS_ 1i_F	GAGGGGCCAAAAGCAAGG	CBS_ 1i_F	GTCACCTACGGGAAGGGACT
CBS 1st intron	CBS_ 1i_2F	CCCTTACCTTGCGAGAGAGG	CBS_ 1i_2R	GTCACCTACGGGAAGGGACT
CBS 3'UTR	CBS_ 3UTR_F	GCAACTGCTGATCGACTTTG	CBS_ 3UTR_R	GTGCTCTGACATGCCTGAAA
CBS 3'UTR	CBS_ 3UTR_2F	ACCCCGTGAAGCAATCATTT	CBS_ 3UTR_2R	TGCCTGAAAATACCATGCAA

Primers used to assay level of mRNA with qPCR and ChIP-qPCR

Hauck et. al. Supplemental Figures and Legends

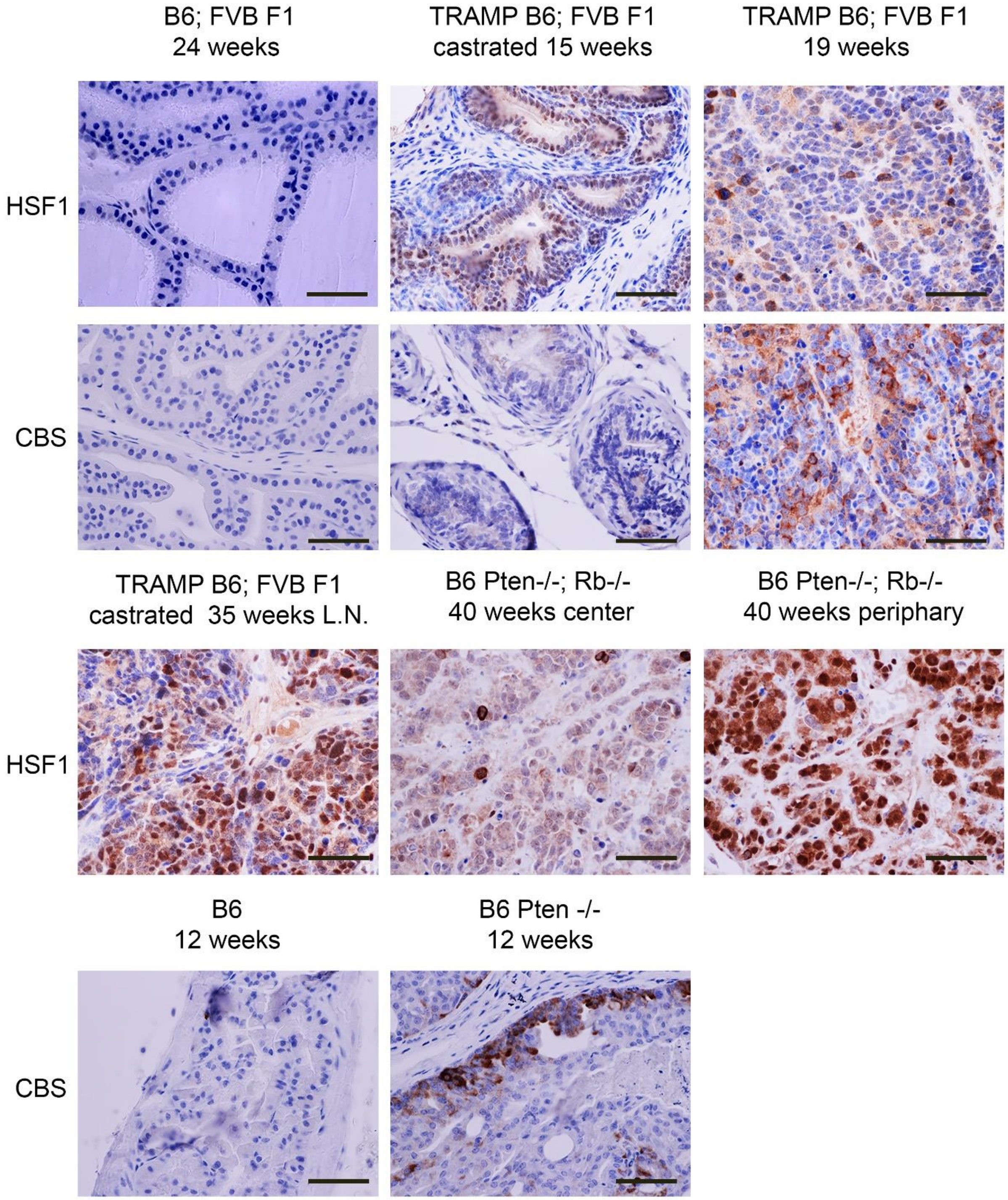

Supplementary Figure 1 HSF1 and CBS levels are increased in mouse PCa models. HSF1 and CBS protein levels are increased in TRAMP FVB F1 mice. HSF1 was increased in lymph node metastasis. Pten; Rb1 double knockout mice had higher HSF1 in the periphery of the tumor where there is often more cell proliferation. CBS protein was detected in 12-week-old Pten knockout mice. Scale bar= 50 µm. LN: lymph node.

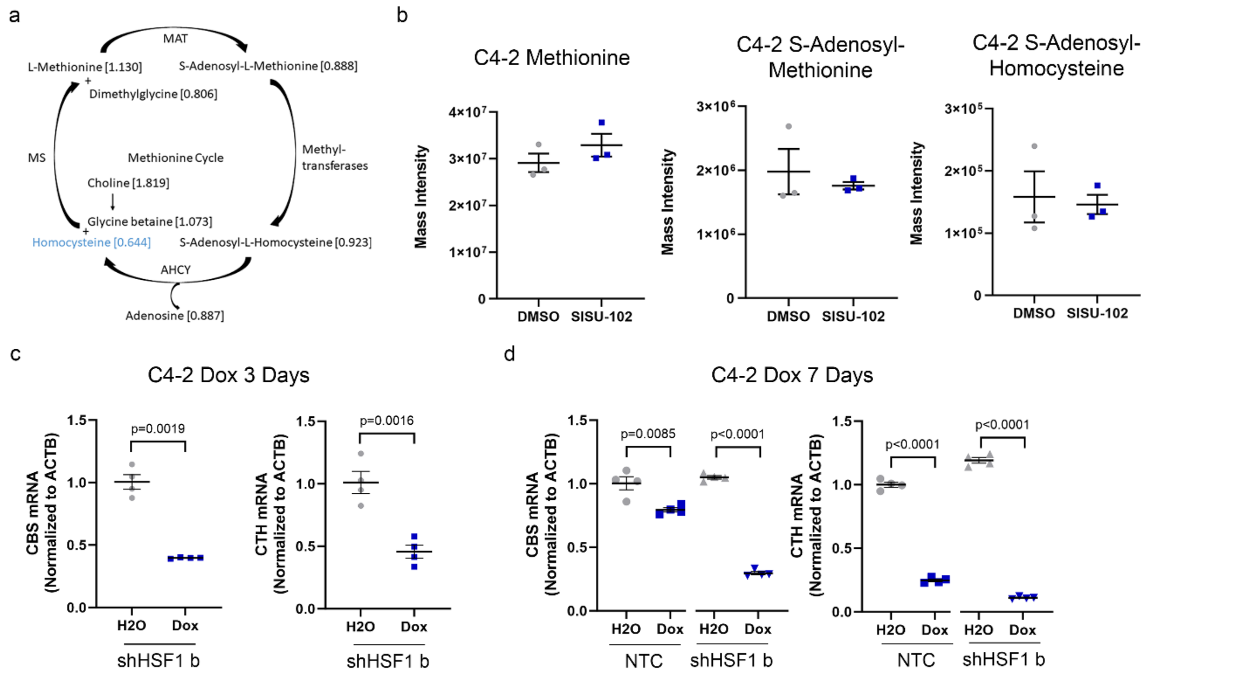

Supplementary Figure 2 Methionine cycle is unaffected by HSF1 inhibition and CBS is reproducibly decreased by shHSF1 but not CTH. (a) Methionine cycle metabolites are shown with red indicating a significant increase and blue indicating a significant decrease in metabolite level. **(b)** SISU-102 treatment of C4-2 cells did not modify the levels of methionine, s-adenosyl-methionine, or s-adenyl-homocysteine (n=3). **c, d** Treatment of C4-2 cells with a different inducible shRNA targeting *HSF1* that was shown in the primary figures with 50 ng per mL doxycycline for 3 days **(c)** decreased CBS levels, but did not affect CTH levels (n=4 technical replicates). **(d)** Doxycycline treatment for 7 days decreased CBS only in the shHSF1 cells, but doxycycline treatment decreased CTH levels in both the NTC and shHSF1 cells (n=4 technical replicates). Mean ± standard error is displayed in dot plots and bar graphs. CTH: cystathionine γ-lyase

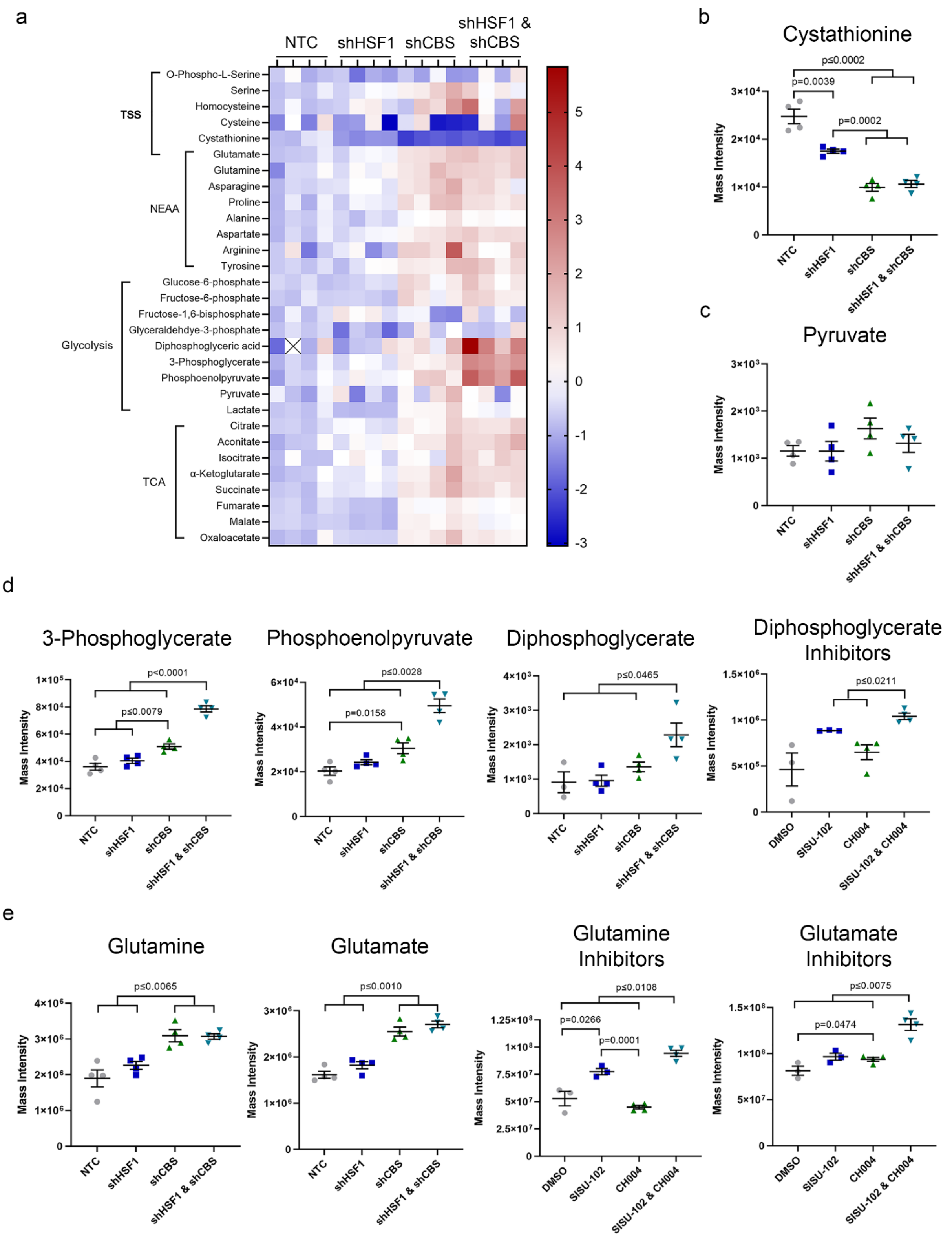

Supplementary Figure 3 *HSF1* and *CBS* knockdown decrease cystathionine and increase precursors to pyruvate, glutamine, and glutamate levels. Transsulfuration metabolites were measured in *HSF1* and *CBS* knockdown C4-2 cells treated with 50 ng per mL doxycycline for 7

days. **(a)** Heat map of metabolites after *HSF1* and *CBS* knockdown in C4-2 cells (n=4). The X in the heat map indicates that one NTC replicate did not have a diphosphoglycerate level detected. **(b)** Cystathionine levels after *HSF1* and *CBS* knockdown. **(c)** Pyruvate levels after HSF1 and *CBS* knockdown. **(d)** Levels of precursors to pyruvate from *HSF1* and *CBS* knockdown or in C4-2 cells treated with 2.5 µM SISU-102 and 10 µM CH004 for 48 hours. **(e)** Glutamine and glutamate levels after *HSF1* and *CBS* knockdown C4-2 cells treated and in C4-2 cells treated with 2.5 µM SISU-102 and 10 µM CH004 for 48 hours. Mean ± standard error is displayed in dot plots. TSS: Transsulfuration pathway, NEAA: non-essential amino acids, and TCA: Tricarboxylic acid cycle.

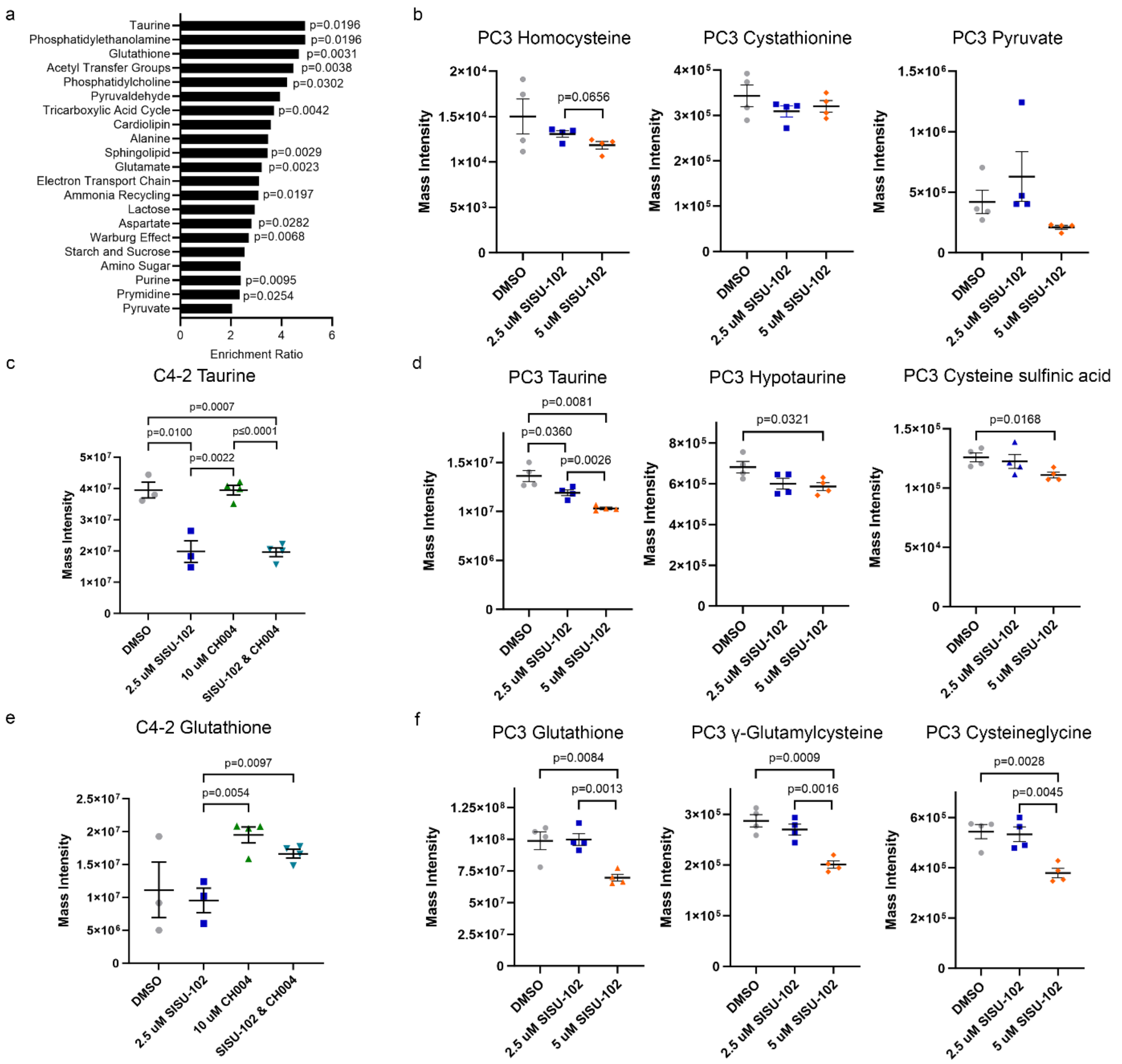

Supplementary Figure 4 Transsulfuration pathway metabolites were unaffected by SISU-102 in PC3 cells, but taurine and glutathione metabolites were decreased.
(a) Homocysteine degradation was not identified from enrichment analysis with MetaboAnalyst of 5 µM SISU-102 treated of PC3 cells, but taurine and glutathione pathway metabolites were affected (n=4). **(b)** Transsulfuration pathway metabolites were unaffected by SISU-102 treated of PC3 cells (n=4). **(c)** C4-2 cells treated with 2.5 µM SISU-102 had decreased levels of taurine (n=4, but 1 replicate was filtered from DMSO and SISU-102 groups due to high variance). **(d)** PC3 cells treated with 5 µM SISU-102 had decreased levels of taurine metabolites (n=4). **(e)** Glutathione levels were unaffected in C4-2 cells treated with 2.5 µM SISU-102 (n=4, but 1 replicate was filtered from DMSO and SISU-102 groups due to high variance). **(f)** Glutathione metabolites were decreased in PC3 cells treated with 5 µM SISU-102 (n=4). Mean ± standard error is displayed in dot plots.

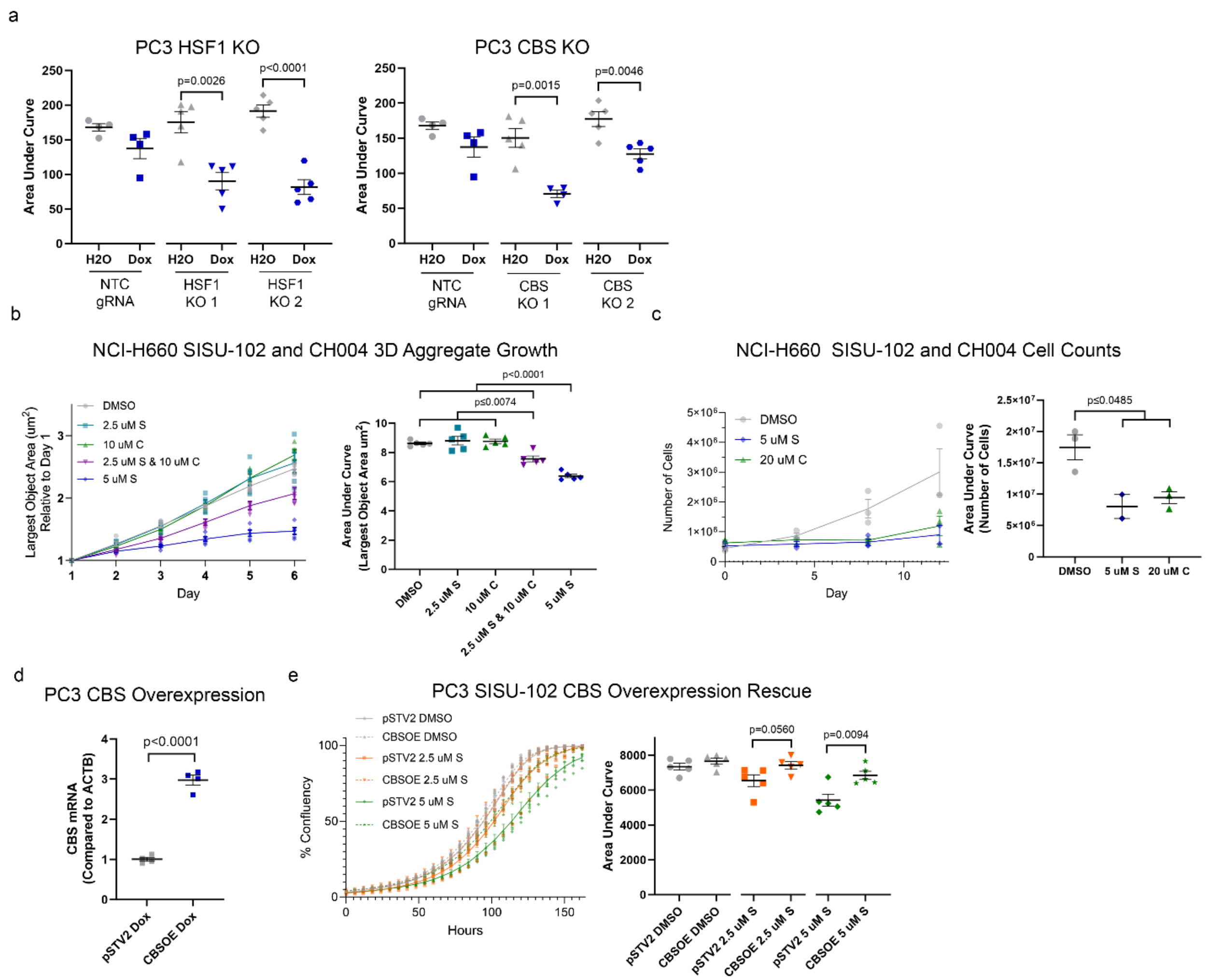

Supplementary Figure 5 PCa growth is modified by HSF1 and CBS. (a) There was a decrease in the growth in inducible Cas9 expressing PC3 cells treated with 2 µg per mL doxycycline for 2 weeks before the IncuCyte growth curve when we targeted *HSF1* and *CBS* with guide RNAs (n=5). **(b)** An additive decrease in growth was observed for NCI-H660 cells treated with the HSF1 inhibitor SISU-102 and the CBS inhibitor CH004 in a 3D aggregate growth assay that analyzed for growth from the beginning of the experiment with area under the curve analysis (n=6). **(c)** There was a significant decrease in the growth of NCI-H660 cells treated with HSF1 inhibitor and a trend of a decrease in growth form the CBS inhibitor in a cell count assay (n=3). **(d)** *CBS* was overexpressed in PC3 cells, and CBS expression was induced with 250 ng per mL doxycycline treatment for 1 week before *CBS* mRNA levels were evaluated (n=4 technical replicates). **(e)** *CBS* overexpression in PC3 cells showed a rescue from 2.5 and 5 µM SISU-102 treatment when pictures were taken every 6 hours in an IncuCyte growth curve (n=5) S: SISU-102 and C: CH004. Mean ± standard error is displayed in dot plots and line graphs.

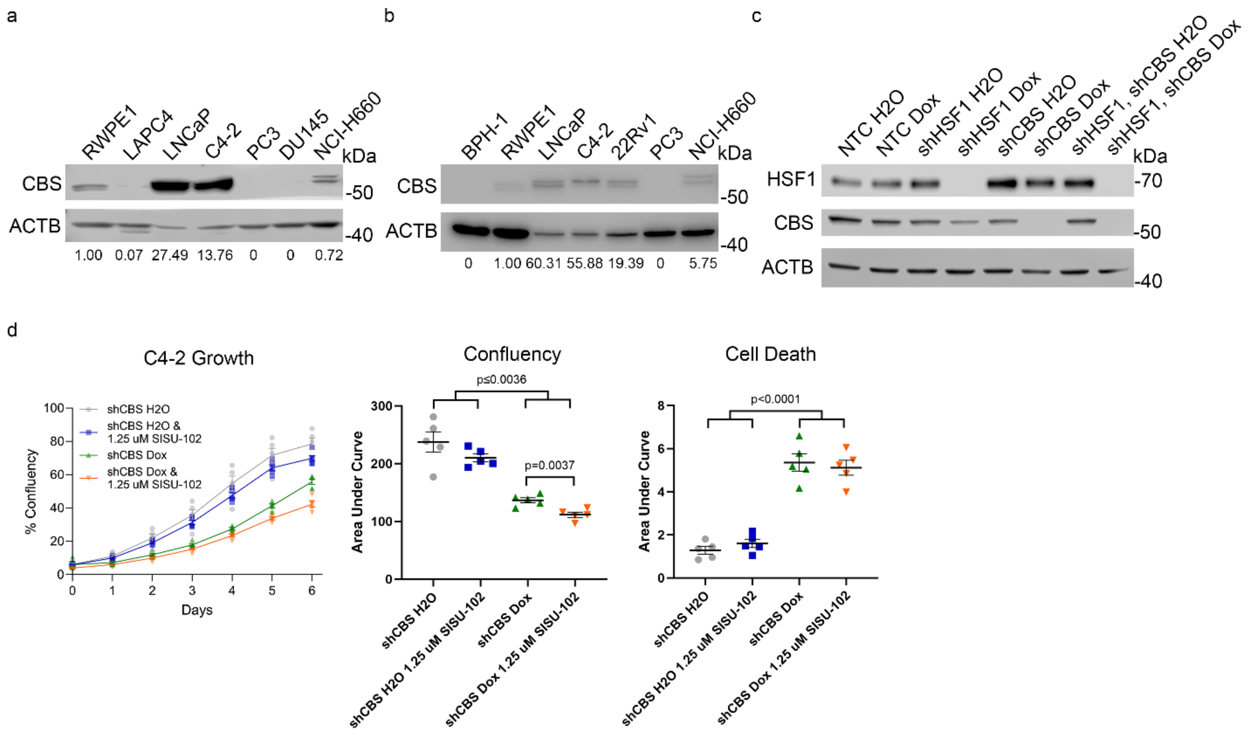

Supplementary Figure 6 CBS levels in PCa lines, Validation of HSF1 and CBS knockdown in C4-2, and HSF1 inhibitor treatment is additive to CBS knockdown in C4-2. (a, b) CBS protein levels were evaluated through western blot analysis with RWPE1 as a control. **(c)** C4-2 with shRNAs for *HSF1* and *CBS* were treated with 50 ng per mL doxycycline for one week. The level of HSF1 and CBS protein was evaluated through western blot analysis. **(d)** C4-2 cells with inducible CBS knockdown were treated for seven days with 50 ng per mL doxycycline. Then, the cells were plated into a growth curve with SISU-102 and doxycycline in the media (n=5). The growth was analyzed in an IncuCyte growth curve with area under the curve analysis of growth from the beginning of the experiment. Cell death was measured with Cytotox green with area under the curve analysis. Mean ± standard error is displayed in line and dot plots.

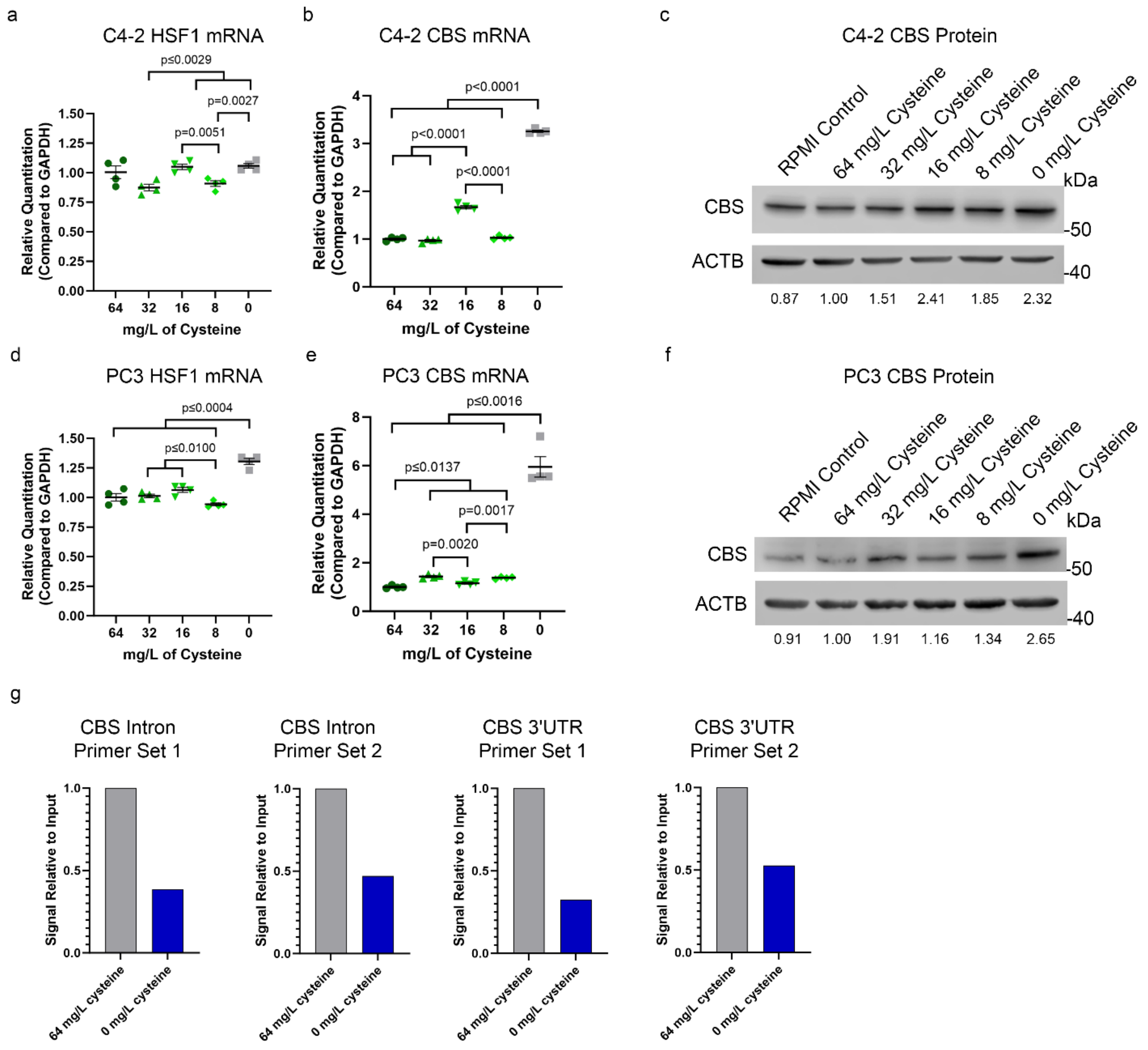

Supplementary Figure 7 Acute cysteine deprivation increases *CBS* mRNA and protein levels independent of HSF1 binding to the *CBS* gene. C4-2 and PC3 cells were deprived of cysteine for 48 hours before *HSF1* and *CBS* mRNA analysis (n=4 technical replicates) **(a, b and d, e)** and CBS protein analysis **(c and f)**. The level of HSF1 binding to the 2 HSF1 binding sites on the *CBS* gene were and measured with ChIP-qPCR after 48 hours of cysteine deprivation (n=4) **(g)**. Mean ± standard error is displayed in the dot plots. The mean is only displayed in bar graphs for ChIP-qPCR experiments.

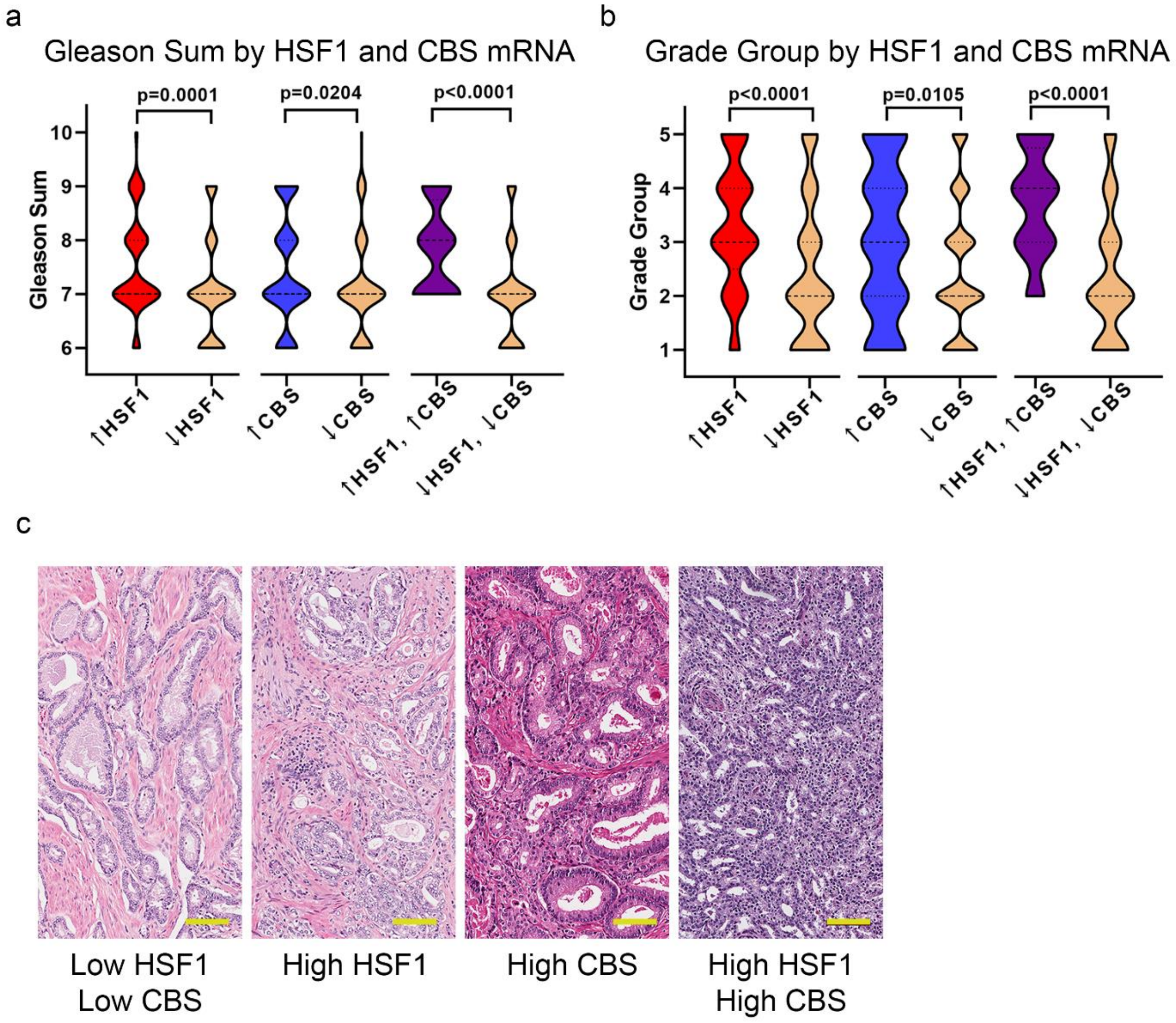

Supplementary Figure 8 HSF1 and CBS predict Gleason score and Grade Group. A-C The ability of *HSF1* and *CBS* mRNA to predict Gleason score and Grade Group was tested in the TCGA database (n=236). **(a)** Gleason score of highest *HSF1* quartile (n=61), highest *CBS* quartile (n=71), or highest *HSF1* and *CBS* quartiles (n=24) versus the corresponding group with the median shown as a dotted line. Groups were analyzed with a Mann-Whitney U test. **(b)** Grade Group of highest *HSF1* quartile (n=61), highest *CBS* quartile (n=71), or highest *HSF1* and *CBS* quartiles (n=24) versus the corresponding group with the median shown as a dotted line. **(c)** Representative hematoxylin and eosin images by *HSF1* and *CBS* group are shown. Scale bar= 100 µm. Groups were analyzed with a Mann-Whitney U test. KO: knockout. Mean ± standard error is displayed in line and bar graphs. Median is shown with the dashed line in the truncated violin plots for Gleason sum and Grade Group with the quartiles shown by the dotted line.

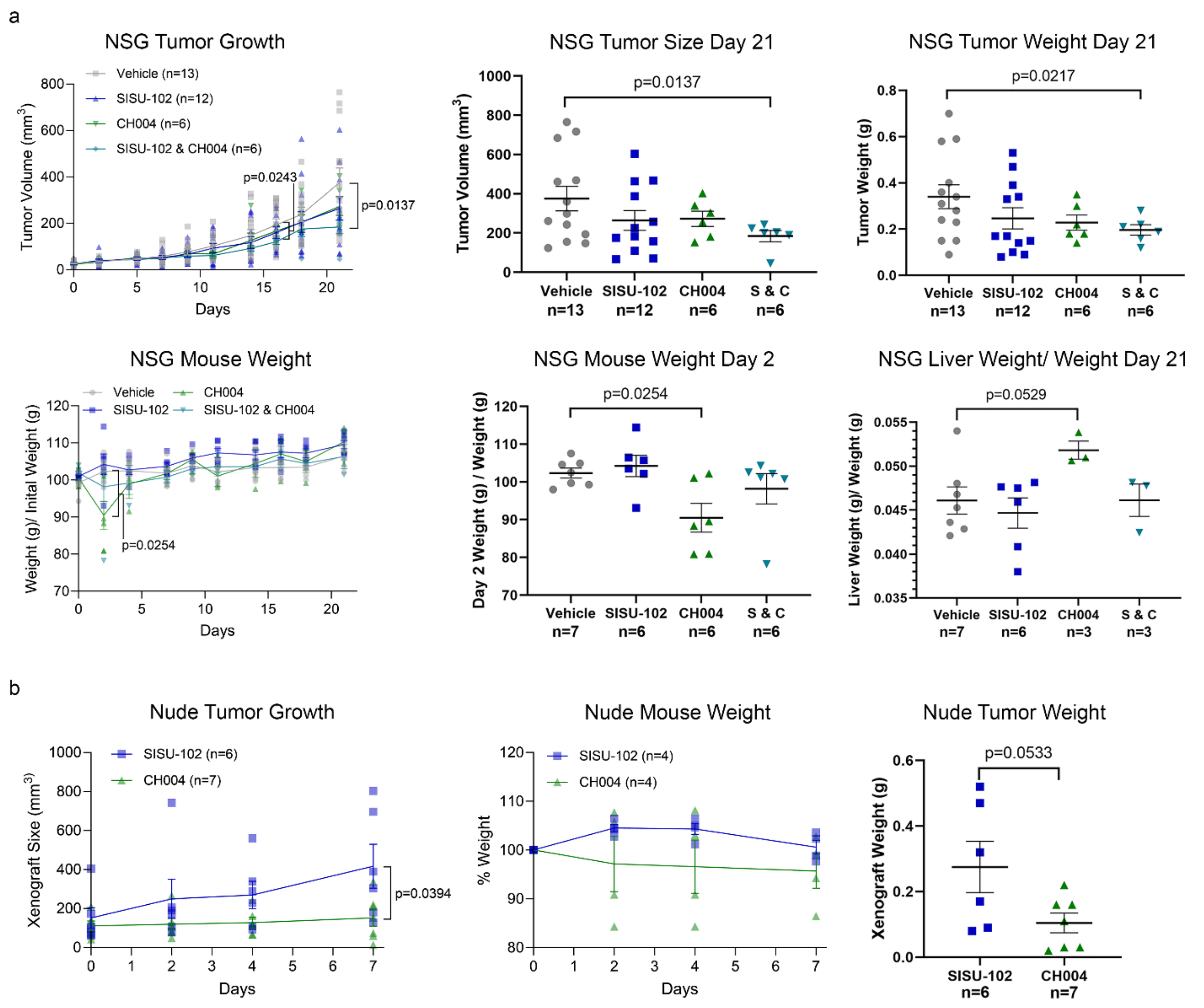

Supplementary Figure 9 The CBS inhibitor CH004 decreases NCI-H660 growth in NSG and nude mice, but CH004 showed toxicity in the animals. NSG mice were injected with 10 mg per kg CH004 in PBS via tail vein, a concentration that did not affect B6 mice after 4 treatments, on day 0 and day 2. Six out of the twelve the mice that were injected with CH004 succumbed to adverse events hours after the second injection, including mice that were also injected with 5 mg per kg SISU-102. However, the mice that fared well after the CH004 treatment and received SISU-102 had smaller tumor size and weight at 21 days (Vehicle n=13, SISU-102 n=12, CH004 n=6, SISU-102 & SISU-102 and CH004 n=6) **(a)**. There were no lesions seen in brain, lung, pancreas, kidney, heart, skeletal muscle, or liver from the CH004 treatment alone or in combination with SISU-102 (data not shown), but mice treated with only CH004 had a trend of an increased liver weight for their body weight. **(b)** We hypothesized that the less immunocompromised athymic nude mice would be less affected by CH004 than NSG mice. However, the nude mice bearing NCI-H660 tumors did not tolerate a single dose of 7.5 or 5 mg per kg CH004. The nude mice did tolerate a single dose of 2.5 mg per kg CH004, and these mice were compared to daily 5 mg per kg SISU-102 in nude mice bearing NCI-H660 xenografts. The single treatment of CH004 decreased NCI-H660 xenograft size in nude mice (SISU-102 n=6, CH004 n=7) and had a trend of decreasing tumor weight while having a modest non-significant decrease on mouse weight (n=4). Mean ± standard error is displayed in line graphs and dot plots.

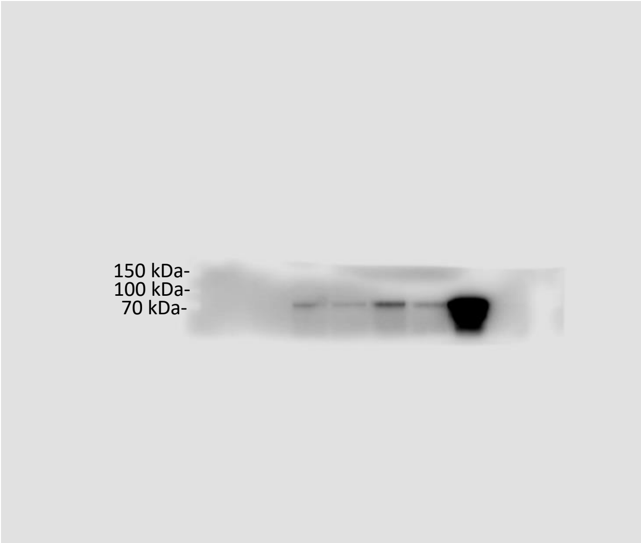

HSF1 in parental prostate cancer cells from Figure 1

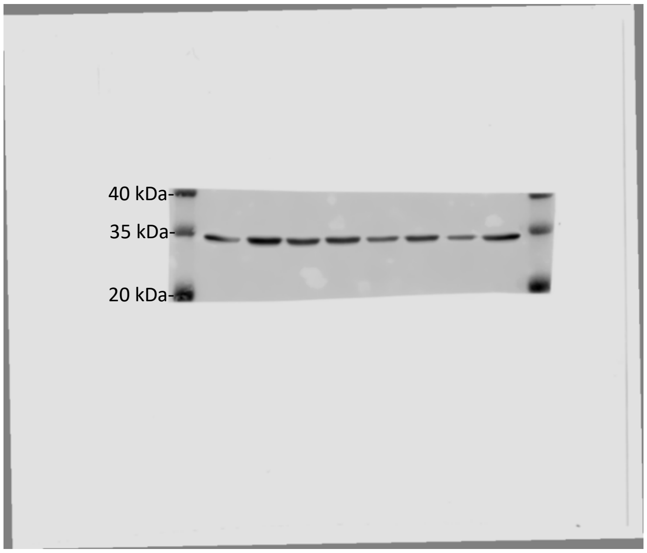

GAPDH in parental prostate cancer cells from Figure 1

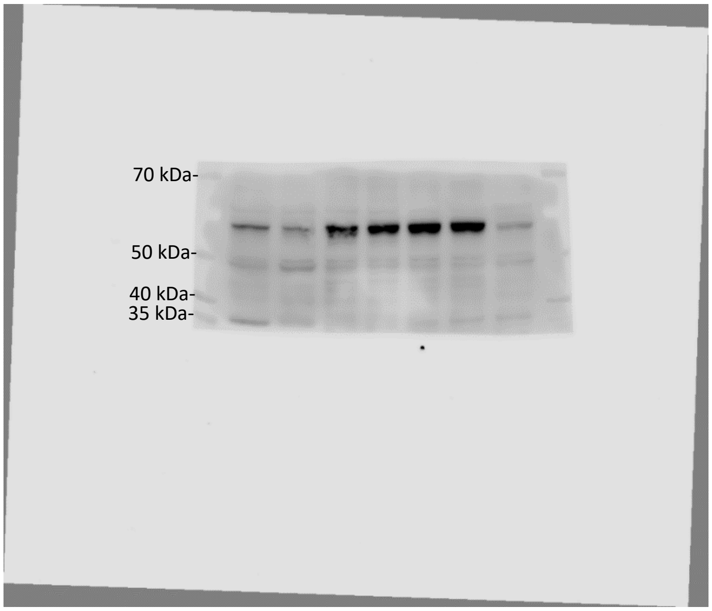

CBS in prostate cancer lines from Figure 3

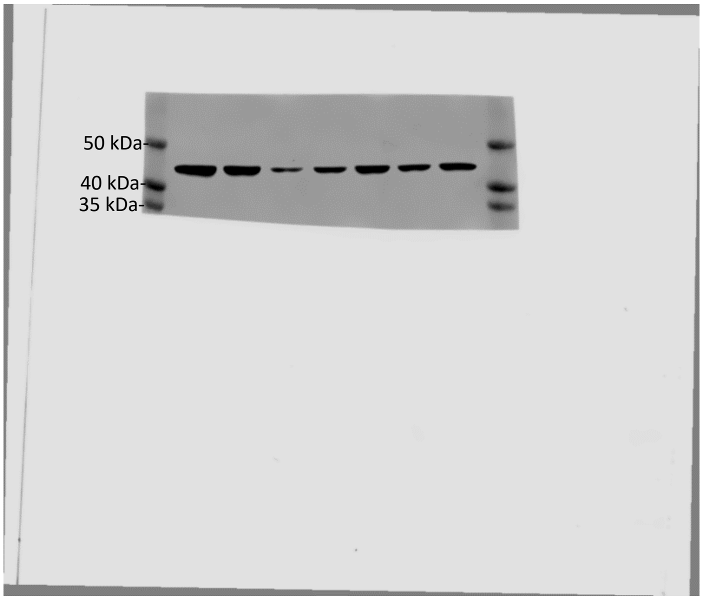

ACTB in prostate cancer lines from Figure 3

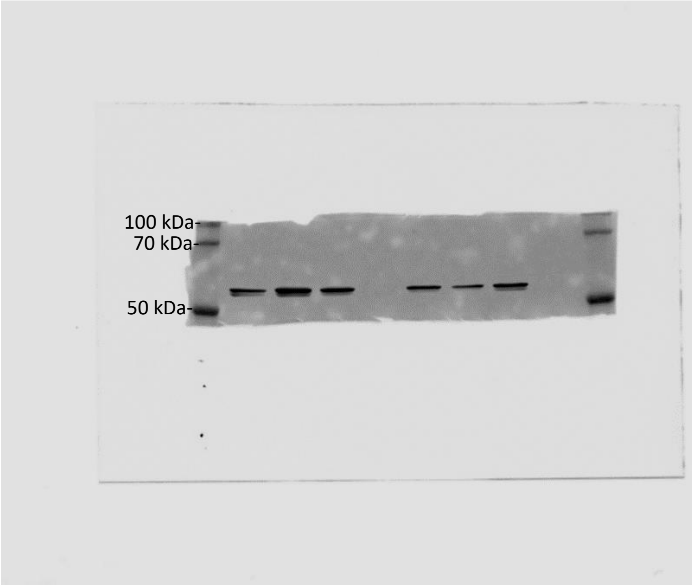

CBS after induction of CBS knockdown from Figure 3 (the other 4 wells were from another inducible CBS shRNA)

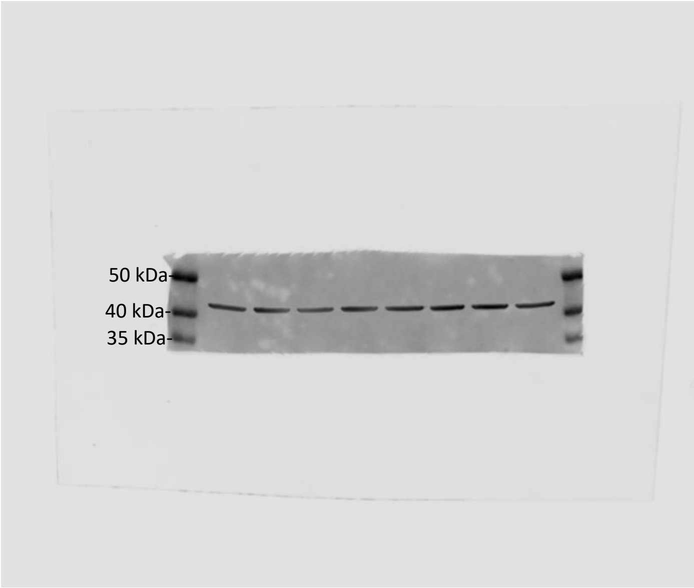

ACTB after induction of CBS knockdown from Figure 3 (the other 4 wells were from another inducible CBS shRNA)

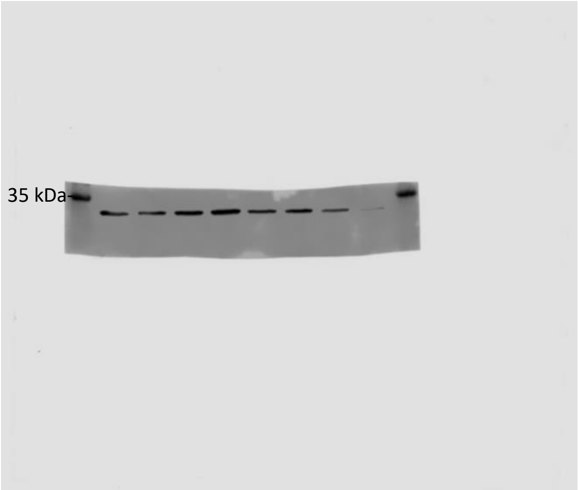

Full length caspase 3 after NCI-H660 was treated with SISU-102 and CH004 from Figure 5

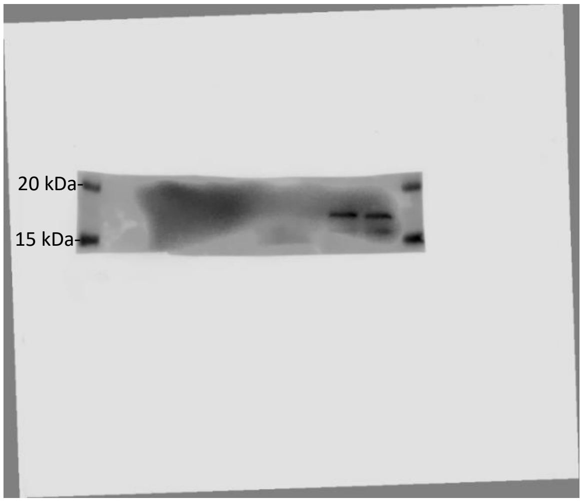

Cleaved caspase 3 after NCI-H660 was treated with SISU-102 and CH004 from Figure 5

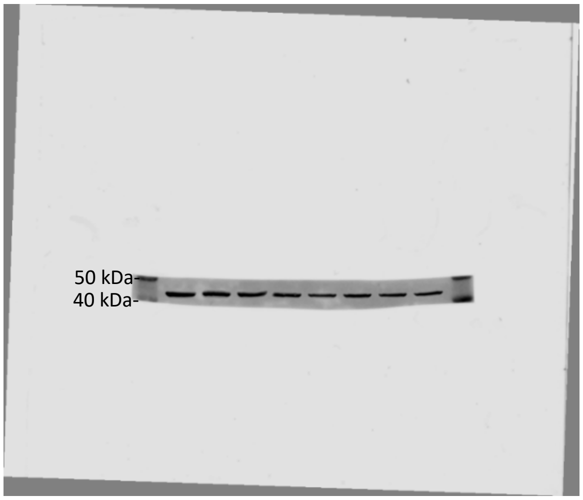

ACTB after NCI-H660 was treated with SISU-102 and CH004 from Figure 5

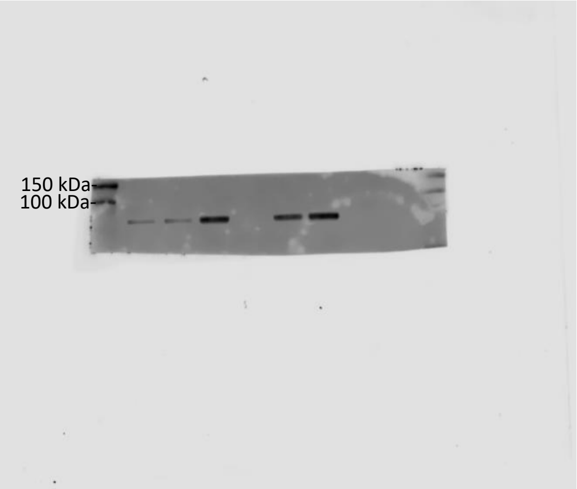

HSF1 after HSF1 knockdown in C4-2 from Figure 6 Lanes 4 and 5 shown in the figure

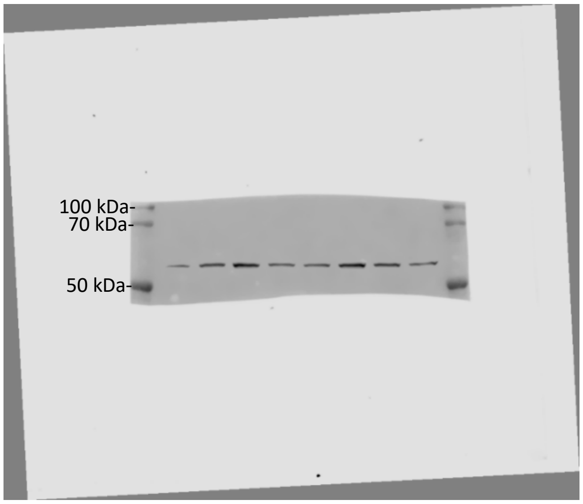

CBS after HSF1 knockdown in C4-2 from Figure 6 Lanes 4 and 5 shown in the figure

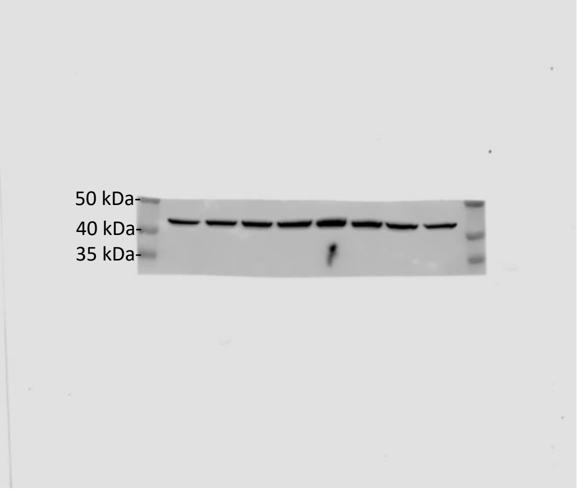

ACTB after HSF1 knockdown in C4-2 from Figure 6 Lanes 4 and 5 shown in the figure

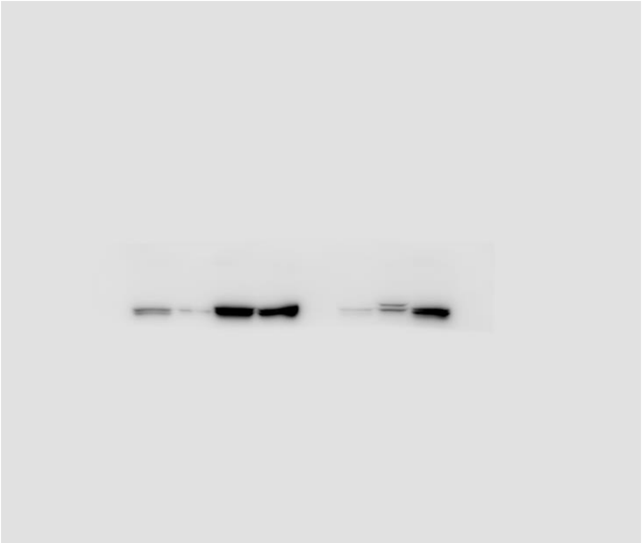

CBS in Prostate Cancer Lines from Supplementary Figure 6a without ladder overlay (Small Cell Neuroendocrine Prostate Cancer cell line MSKCC-EF1 in lane 9 had very high levels of CBS protein, but was not evaluated in the paper)

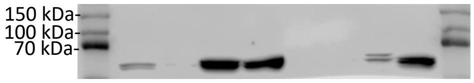

CBS in Prostate Cancer Lines from Supplementary Figure 6a with ladder overlay that was cropped to just have the membrane in the image (Small Cell Neuroendocrine Prostate Cancer cell line MSKCC-EF1 in lane 9 had very high levels of CBS protein, but was not evaluated in the paper)

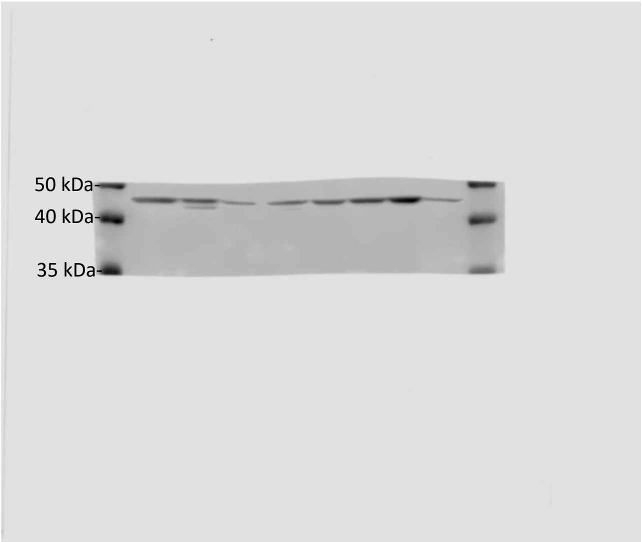

ACTB in Prostate Cancer Lines from Supplementary Figure 6a (Small Cell Neuroendocrine Prostate Cancer cell line MSKCC-EF1 in lane 9 had very high levels of CBS protein, but was not evaluated in the paper)

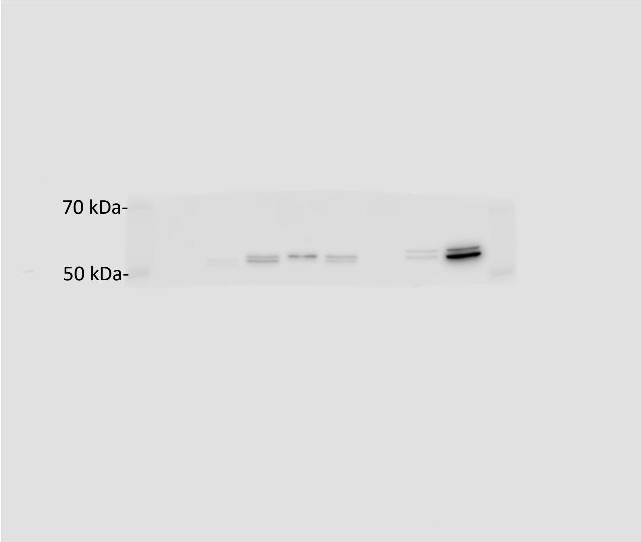

CBS in Prostate Cancer Lines from Supplementary Figure 6b (Small Cell Neuroendocrine Prostate Cancer cell line MSKCC-EF1 in lane 9 had very high levels of CBS protein, but was not evaluated in the paper)

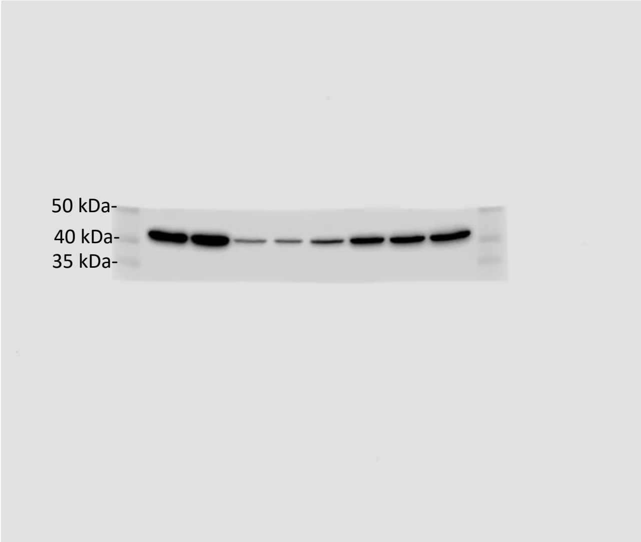

ACTB in Prostate Cancer Lines from Supplementary Figure 6b (Small Cell Neuroendocrine Prostate Cancer cell line MSKCC-EF1 in lane 9 had very high levels of CBS protein, but was not evaluated in the paper)

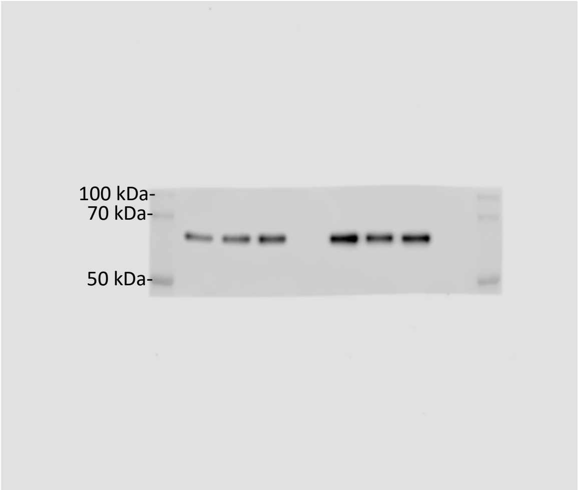

HSF1 C4-2 HSF1 and CBS inducible knockout from Supplementary Figure 6c

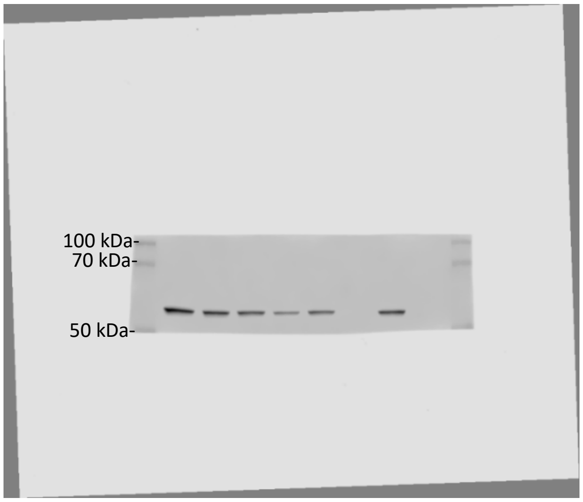

CBS C4-2 HSF1 and CBS inducible knockout from Supplementary Figure 6c

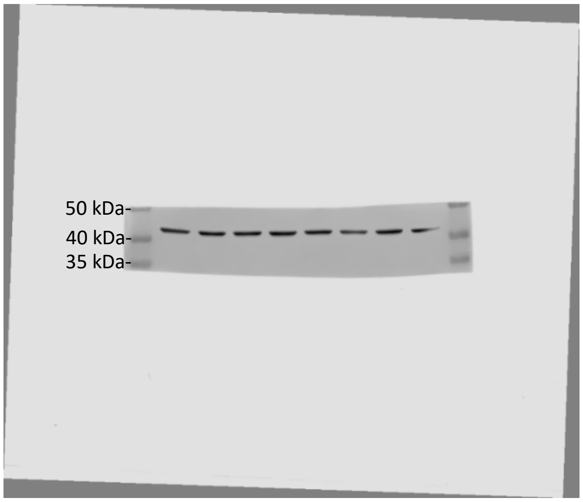

ACTB C4-2 HSF1 and CBS inducible knockout from Supplementary Figure 6c

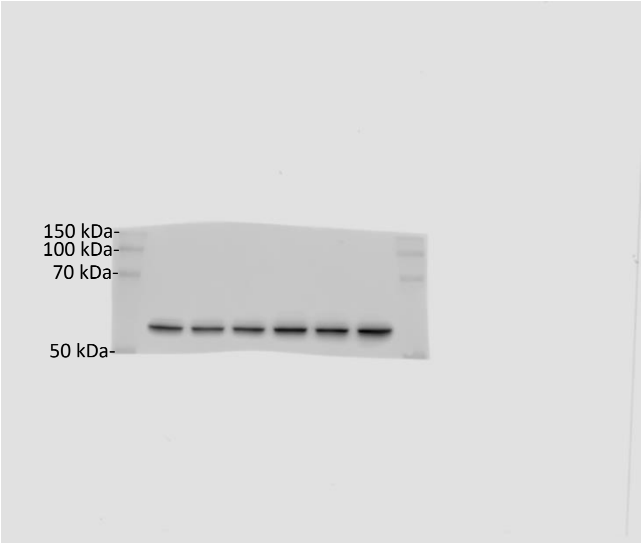

CBS C4-2 with 48 hours of cysteine deprivation from Supplementary Figure 7c

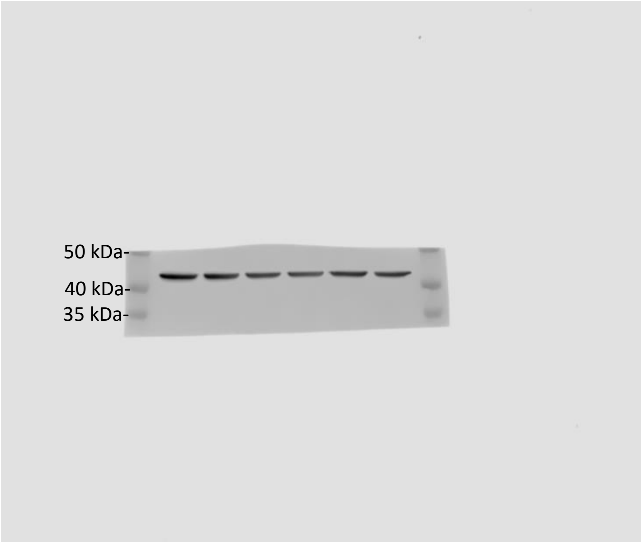

ACTB C4-2 with 48 hours of cysteine deprivation from Supplementary Figure 7c

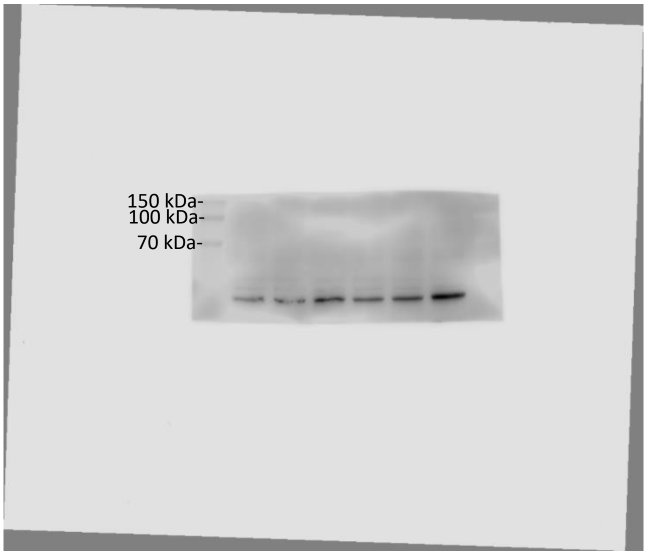

CBS PC3 with 48 hours of cysteine deprivation from Supplementary Figure 7f

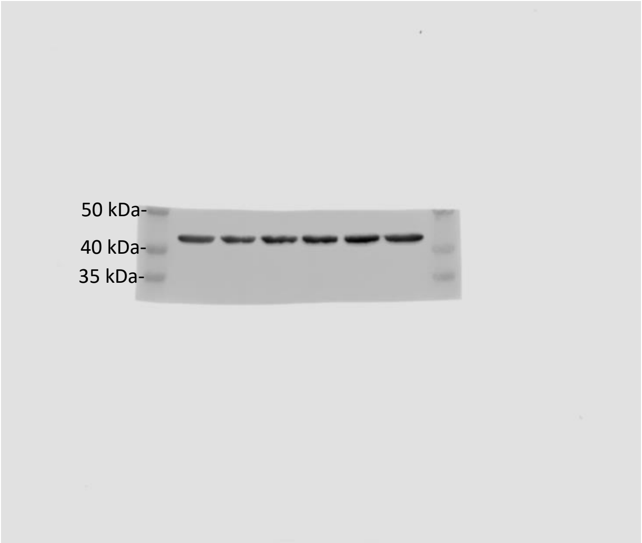

ACTB with 48 hours of cysteine deprivation from Supplementary Figure 7f

Supplementary Figure 10 Unedited Western Blots Western blot membranes were developed by Chemiluminescent Substrate (Thermo Fisher) for HRP conjugated antibody detection and exposed by Odyssey Imaging Systems (LI-COR). Overlay with the 700 nm channel was done to visualize the protein ladder for all unedited blots accept the blot evaluating CBS in Prostate Cancer Lines from Supplementary Figure 6a. However, an image with the 700 nm overlay that was cropped to just have the membrane in the image is provided for comparison.
