# Supplementary material for: Heat shock factor 1 directly regulates transsulfuration pathway to promote prostate cancer proliferation and survival
Source: Commun Biol. 2024 Jan 3;7:9. doi: 10.1038/s42003-023-05727-9 (PMC10764307; doi:10.1038/s42003-023-05727-9)
Supplement: Supplementary file 5 — Reporting Summary [file 42003_2023_5727_MOESM5_ESM.pdf]

nature portfolio

Corresponding author(s): Jiaoti Huang

Last updated by author(s): Dec 12, 2023

Reporting Summary

Nature Portfolio wishes to improve the reproducibility of the work that we publish. This form provides structure for consistency and transparency in reporting. For further information on Nature Portfolio policies, see our Editorial Policies and the Editorial Policy Checklist.

Statistics

For all statistical analyses, confirm that the following items are present in the figure legend, table legend, main text, or Methods section.

n/a	Confirmed	
☐	☒	The exact sample size (*n*) for each experimental group/condition, given as a discrete number and unit of measurement
☐	☒	A statement on whether measurements were taken from distinct samples or whether the same sample was measured repeatedly
☐	☒	The statistical test(s) used AND whether they are one- or two-sided<br>*Only common tests should be described solely by name; describe more complex techniques in the Methods section.*
☐	☒	A description of all covariates tested
☐	☒	A description of any assumptions or corrections, such as tests of normality and adjustment for multiple comparisons
☐	☒	A full description of the statistical parameters including central tendency (e.g. means) or other basic estimates (e.g. regression coefficient) AND variation (e.g. standard deviation) or associated estimates of uncertainty (e.g. confidence intervals)
☒	☐	For null hypothesis testing, the test statistic (e.g. *F*, *t*, *r*) with confidence intervals, effect sizes, degrees of freedom and *P* value noted<br>*Give P values as exact values whenever suitable.*
☒	☐	For Bayesian analysis, information on the choice of priors and Markov chain Monte Carlo settings
☒	☐	For hierarchical and complex designs, identification of the appropriate level for tests and full reporting of outcomes
☒	☐	Estimates of effect sizes (e.g. Cohen's *d*, Pearson's *r*), indicating how they were calculated

Our web collection on statistics for biologists contains articles on many of the points above.

Software and code

Policy information about availability of computer code

Data collection	N/A
Data analysis	N/A

For manuscripts utilizing custom algorithms or software that are central to the research but not yet described in published literature, software must be made available to editors and reviewers. We strongly encourage code deposition in a community repository (e.g. GitHub). See the Nature Portfolio guidelines for submitting code & software for further information.

Data

Policy information about availability of data

All manuscripts must include a data availability statement. This statement should provide the following information, where applicable:

- Accession codes, unique identifiers, or web links for publicly available datasets
- A description of any restrictions on data availability
- For clinical datasets or third party data, please ensure that the statement adheres to our policy

The source data behind the graphs in the paper are available in Supplementary Data. The data generated during and/or analyzed during the current study is being stored in a LabArchives Electronic Lab Notebook, and they are available from the corresponding author Jiaoti Huang on reasonable request. Source databases to evaluate the levels of HSF1 and CBS across PCa patient types in this study include TCGA-PRAD https://portal.gdc.cancer.gov/projects/TCGA-PRAD accessed 5/14/2020, Grasso 2012 [GSE35988] https://www.ncbi.nlm.nih.gov/geo/query/acc.cgi?acc=GSE35988 and [GSE35988] https://www.ncbi.nlm.nih.gov/geo/query/

acc.cgi?acc=GSE35988, Genomic Characterization of Metastatic Castration Resistant Prostate Cancer [phs001648.v1.p1] access needs to be requested from the original authors to access these data https://www.ncbi.nlm.nih.gov/projects/gap/cgi-bin/study.cgi?study_id=phs001648.v1.p1, and Molecular Basis of Neuroendocrine Prostate Cancer (Trento/Cornell/Broad 2015) [phs000909.v1.p1] access needs to be requested from the original authors to access these data https://www.ncbi.nlm.nih.gov/projects/gap/cgi-bin/study.cgi?study_id=phs000909.v1.p1. The source database to evaluate the survival of mCRPC patients by the levels of HSF1 mRNA was Genomic Characterization of Metastatic Castration Resistant Prostate Cancer [phs001648.v1.p1] access needs to be requested from the original authors to access these data https://www.ncbi.nlm.nih.gov/projects/gap/cgi-bin/study.cgi?study_id=phs001648.v1.p1. The source database to evaluate Gleason Sum and Grade Group for PCa patients was TCGA-PRAD https://portal.gdc.cancer.gov/projects/TCGA-PRAD accessed 5/14/2020.

Human research participants

Policy information about studies involving human research participants and Sex and Gender in Research.

Reporting on sex and gender: Only male participants were included because only men get prostate cancer.

Population characteristics: Benign and adenocarcinoma prostatectomy specimens from (n = 40) patients were used to make the tissue microarrays including adjacent benign tissue from adenocarcinoma patients. CRPC tissue microarrays samples were obtained through transurethral resection patients who received hormonal therapy, rather than prostatectomy, and had urinary obstruction due to tumor recurrence. SCNC tissue microarrays were constructed from (n =17) primary SCNC cases.

Recruitment: All samples were collected from patients with informed consent who were treated at Duke University.

Ethics oversight: All related procedures were performed with the approval of the internal review and ethics boards of Duke University IRB approval Pro00080721.

Note that full information on the approval of the study protocol must also be provided in the manuscript.

Field-specific reporting

Please select the one below that is the best fit for your research. If you are not sure, read the appropriate sections before making your selection.

☒ Life sciences ☐ Behavioural & social sciences ☐ Ecological, evolutionary & environmental sciences

For a reference copy of the document with all sections, see nature.com/documents/nr-reporting-summary-flat.pdf

Life sciences study design

All studies must disclose on these points even when the disclosure is negative.

Sample size: The sample size for the SISU-102 treatment xenograft with NCI-H660 xenograft study was estimated by treatment of C4-2 with the HSF1 inhibtior SISU-102. We calculated that our study was powered to test the primary hypothesis that there is a difference in tumor size between HSF1 inhibitor and vehicle treatment from analysis of 21 days of HSF1 inhibitor treatment in a C4-2 xenograft model. With 10 mice per group and up to 10% attrition, the two-sample t-test with a type I error of 0.01 will have 90% power to detect a minimum effect size of about 1.95 between the two groups (Gpower 3.1). The groups for the NCI-H660 SISU-102 xenograft study were Vehicle n=9 and SISU-102 n=10. However, we were using both HSF1 and CBS knockout in NCI-H660, and we had shown that there was an additive effect of HSF1 and CBS inhibition and knockout. Based on these data, we chose 3 mice per group with 2 tumors each to make 6 tumors total. This n number was chosen with the goal of keeping the number of mice used low. All in vitro experiments were performed with three or more biological replicates. IncuCyte growth curves had 5 to 6 biological replicates. All n number represent biological replicates unless explicitly stated in a figure legend. All qPCR and ChIP-qPCR experiments had 3 to 4 technical replicates.

Data exclusions: High level of variance of qPCR technical replicates, wells of growth curves replicates, and metabolite profiling replicates resulted in exclusion from analysis. qPCR results with high Ct values for endogenous controls were excluded.

Replication: All growth curves were repeated at least twice and had similar results. Representative western blots shown in figures were repeated three times independently with similar results.

Randomization: Mice were randomized to ensure that the average weight for each group was not different. Only male mice were used in the studies because only men develop prostate cancer.

Blinding: JSH was not blinded to the mice in the experiments, but the scientists measuring the xenograft size were blinded to the treatment groups.

Reporting for specific materials, systems and methods

We require information from authors about some types of materials, experimental systems and methods used in many studies. Here, indicate whether each material, system or method listed is relevant to your study. If you are not sure if a list item applies to your research, read the appropriate section before selecting a response.

Materials & experimental systems

n/a	Involved in the study
☐	☒ Antibodies
☐	☒ Eukaryotic cell lines
☒	☐ Palaeontology and archaeology
☐	☒ Animals and other organisms
☐	☒ Clinical data
☒	☐ Dual use research of concern

Methods

n/a	Involved in the study
☒	☐ ChIP-seq
☒	☐ Flow cytometry
☒	☐ MRI-based neuroimaging

Antibodies

Antibodies used

Western blot primary: HSF1 (Enzo 10H8 #ADI-SPA-950-D 1: 1000), GAPDH (Cell Signaling 14C10 #2118 1:1000), CBS (Sigma 3E1 #WH0000875M1 1 ug per mL), β-actin (Cell Signaling 13E5 #4970 1:1000), Caspase 3 (Cell Signaling #9662 1:1000), and cleaved Caspase 3 (Cell Signaling #9661 1:1000).
Western blot secondary: Goat anti-rat HRP (Invitrogen # 31470 1:10000), Goat anti-mouse HRP (BioRad #1721011 1.34:4000), and Goat anti-rabbit HRP (BioRad # 1706515 1.34:4000)
ChIP-qPCR: HSF1 (Abcam EP1710Y #ab52757) or HA tag (Abcam #ab9110) at 4 ug
IHC: HSF1 (AbCam #ab57757 at 1:200) and CBS (Sigma 3E1 #WH0000875M1 at 200 ng per mL)

Validation

Western blot primary
• HSF1 (Enzo 10H8 #ADI-SPA-950-D 1: 1000)
o Validation: IMMUNOGEN Recombinant mouse HSF1, UNIPROT ID P38532, GENE/PROTEIN IDENTIFIER NM_008296 (RefSeq), SOURCE Purified from ascites, SPECIES REACTIVITY Human, Mouse, Rat Monkey, Rabbit, APPLICATIONS IHC (PS) and WB RECOMMENDED, and DILUTIONS/CONDITIONS Western Blot (1:1,000, colorimetric) Source Rat
• GAPDH (Cell Signaling 14C10 #2118 1:1000)
o Validation: REACTIVITY Human, Mouse, Rat, Monkey, Bovine, Pig, SENSITIVITY Endogenous, MW (kDa) 37, Source/Isotype Rabbit, Western Blotting 1:1000, and Monoclonal antibody is produced by immunizing animals with a synthetic peptide corresponding to residues surrounding Lys260 of human GAPDH protein.
• CBS (Sigma 3E1 #WH0000875M1 1 ug per mL)
o Validation: purified immunoglobulin, biological source mouse, unconjugated, purified immunoglobulin, species reactivity human, technique(s) immunohistochemistry (formalin-fixed, paraffin-embedded sections): suitable immunoprecipitation (IP): suitable indirect ELISA: suitable western blot: 1-5 µg/mL, GenBank® accession no. NM_000071, and UniProt accession no. P35520
• β-actin (Cell Signaling 13E5 #4970 1:1000)
o Validation: REACTIVITY Human, Mouse, Rat, Monkey, Bovine, Pig, SENSITIVITY Endogenous, MW (kDa) 45, Source/Isotype Rabbit IgG, Western Blotting 1:1000, and β-Actin (13E5) Rabbit mAb detects endogenous levels of total β-actin protein. Depsite the high sequence identity between the cytoplasmic actin isoforms, β-actin and cytoplasmic γ-actin, β-Actin (13E5) Rabbit mAb #4970 does not cross-react with cytoplasmic γ-actin, or any other actin isoforms.
• Caspase 3 (Cell Signaling #9662 1:1000)
o Validation: REACTIVITY Human, Mouse, Rat, Monkey, SENSITIVITY Endogenous, MW (kDa) 17, 19, 35, SOURCE Rabbit, and Caspase-3 Antibody detects endogenous levels of full length caspase-3 (35 kDa) and the large fragment of caspase-3 resulting from cleavage (17 kDa).
• Cleaved Caspase 3 (Cell Signaling #9661 1:1000)
o Validation: REACTIVITY Human, Mouse, Rat, Monkey, SENSITIVITY Endogenous, MW (kDa) 17, 19, SOURCE Rabbit, and Cleaved Caspase-3 (Asp175) Antibody detects endogenous levels of the large fragment (17/19 kDa) of activated caspase-3 resulting from cleavage adjacent to Asp175. This antibody does not recognize full length caspase-3 or other cleaved caspases. This antibody detects non-specific caspase substrates by western blot. Non-specific labeling may be observed by immunofluorescence in specific sub-types of healthy cells in fixed-frozen tissues (e.g. pancreatic alpha-cells). Nuclear background may be observed in rat and monkey samples.
Western blot secondary
• Goat anti-rat HRP (Invitrogen # 31470 1:10000)
o Validation: Species Reactivity Rat, Host/Isotype Goat / IgG, Class Polyclonal, Type Secondary Antibody, Conjugate HRP, Western Blot (WB) 1:5,000-1:200,000 10 Publications, and Antibody Specificity: This antibody reacts with the heavy chains of rat IgG and with the light chains common to most rat immunoglobulins, based on immunoelectrophoresis. No antibody was detected against non-immunoglobulin serum proteins. However, this antibody may cross-react with immunoglobulins from other species
• Goat anti-mouse HRP (BioRad #1721011 1.34:4000)
o Validation: 2 ml, EIA-grade, enzyme-antibody conjugate
• Goat anti-rabbit HRP (BioRad # 1706515 1.34:4000)
o Validation: 2 ml, blotting-grade horseradish peroxidase secondary antibody conjugate
ChIP-qPCR
• HSF1 (Abcam EP1710Y #ab52757)
o Validation: Rabbit monoclonal [EP1710Y] to HSF1 - ChIP Grade, Host species Rabbit, Reacts with: Mouse, Human, Immunogen Synthetic peptide within Human HSF1 aa 450 to the C-terminus (C terminal). The exact sequence is proprietary., Positive control WB: K562 HAP1 and HeLa whole cell lysate (ab150035). ICC/IF: MCF-7 cells. Flow Cyt (intra): HeLa cells. IHC-P: Human ovarian carcinoma tissue; Mouse testis and colon tissue., General notes: This product is a recombinant monoclonal antibody, which offers several advantages including: High batch-to-batch consistency and reproducibility, Improved sensitivity and specificity, Long-term security of supply, and Animal-free production
• HA tag (Abcam #ab9110)
o Validation: Rabbit polyclonal to HA tag - ChIP Grade, Host species Rabbit, Specificity ELISA: The anti HA diluted 1:70.000 gave an O.D.=1.0 in a 15 minute reaction against peptide conjugated with a different carrier than used for anti peptide purification. HRP conjugated Goat anti rabbit IgG was used and TMB was the substrate., Immunogen Synthetic peptide corresponding to Influenza A HA tag conjugated to keyhole limpet haemocyanin. Influenza hemagglutinin-HA-epitope, and Positive control WB: 293FT cells

transfected with 15kDa HA tagged Vpr (an HIV1 accessory protein). IP: Nuclear lysate of HEK-293T cells transiently expressing HA-tagged protein. ICC/IF: U-2 cells. Mouse olineu cells. ChIP: Xenopus laevis oocytes were injected with mRNA for HA-tagged human BORIS.
IHC
• HSF1 (Abcam EP1710Y AbCam #ab52757 at 1:200)
o Validation: Validation: Rabbit monoclonal [EP1710Y] to HSF1 - ChIP Grade, IHC-Paraffin 1/250, Host species Rabbit, Reacts with: Mouse, Human, Immunogen Synthetic peptide within Human HSF1 aa 450 to the C-terminus (C terminal). The exact sequence is proprietary., Positive control WB: K562 HAP1 and HeLa whole cell lysate (ab150035). ICC/IF: MCF-7 cells. Flow Cyt (intra): HeLa cells. IHC-P: Human ovarian carcinoma tissue; Mouse testis and colon tissue., General notes: This product is a recombinant monoclonal antibody, which offers several advantages including: High batch-to-batch consistency and reproducibility, Improved sensitivity and specificity, Long-term security of supply, and Animal-free production
• CBS (Sigma 3E1 #WH0000875M1 at 200 ng per mL)
o Validation: purified immunoglobulin, biological source mouse, unconjugated, purified immunoglobulin, species reactivity human, technique(s) immunohistochemistry (formalin-fixed, paraffin-embedded sections): suitable immunoprecipitation (IP): suitable indirect ELISA: suitable western blot: 1-5 µg/mL, GenBank® accession no. NM_000071, and UniProt accession no. P35520

Eukaryotic cell lines

Policy information about cell lines and Sex and Gender in Research

Cell line source(s): Human PCa lines, LNCAP, C4-2, PC3, DU145, 22Rv1, NCI-H660, and immortalized prostatic epithelial cell line RWPE1 were purchased from ATCC. Benign prostatic hyperplasia BPH-1 cells were purchased form Millipore Sigma. Human PCa line LAPC4 was obtained from the laboratory of Charles Sawyers and CWR-R1 was obtained from the laboratory of Elizabeth M. Wilson. All cell lines were derived from human males.

Authentication: The purchase of cell lines from ATCC or Millipore Sigma sufficed as our validation.

Mycoplasma contamination: Cell lines were not routinely tested for mycoplasma contamination

Commonly misidentified lines (See ICLAC register): N/A

Animals and other research organisms

Policy information about studies involving animals; ARRIVE guidelines recommended for reporting animal research, and Sex and Gender in Research

Laboratory animals: NOD-scid IL2Rgammanull (NSG) mice and nu/nu (nude) mice

Wild animals: N/A

Reporting on sex: Only male mice were used in the studies because only men develop prostate cancer.

Field-collected samples: N/A

Ethics oversight: All animal studies were approved by the Institutional Animal Care and Use Committee (IACUC) of Duke University (A055-22-03) and Department of Defense Animal Care and use Review Office.

Note that full information on the approval of the study protocol must also be provided in the manuscript.

Clinical data

Policy information about clinical studies
All manuscripts should comply with the ICMJE guidelines for publication of clinical research and a completed CONSORT checklist must be included with all submissions.

Clinical trial registration: All related procedures were performed with the approval of the internal review and ethics boards of Duke University IRB approval Pro00080721, but were not part of a clinical trial.

Study protocol: *Note where the full trial protocol can be accessed OR if not available, explain why.*

Data collection: *Describe the settings and locales of data collection, noting the time periods of recruitment and data collection.*

Outcomes: *Describe how you pre-defined primary and secondary outcome measures and how you assessed these measures.*
